# The potential of *Pseudomonas* spp. as sustainable bioinoculants for enhancing maize growth and integrated management of drought and *Fusarium verticillioides* stress

**DOI:** 10.1007/s00425-025-04906-8

**Published:** 2026-01-28

**Authors:** Khethiwe Ndlazi, Siyabonga Ntshalintshali, Lungelo Buthelezi, Ashwil Klein, Marshall Keyster, Mbukeni Nkomo, Arun Gokul

**Affiliations:** 1 Department of Agriculture, University of Zululand, Main Road, P/Bag X1001, Kwadlangezwa, 3886 South Africa; 2 Plant Biotechnology Laboratory, School of Science and Agriculture, Westville Campus, University of KwaZulu-Natal, University Road, Durban, 4000 South Africa; 3 School of Biology and Environmental Sciences, Faculty of Agriculture and Natural Sciences, University of Mpumalanga, Mbombela, 1200 South Africa; 4 Plant Omics Laboratory, Department of Biotechnology, University of the Western Cape, Robert Sobukwe Road, Bellville, 7530 South Africa; 5 Environmental Biotechnology Laboratory, Department of Biotechnology, University of the Western Cape, Robert Sobukwe Road, Bellville, 7530 South Africa

**Keywords:** Adaptation mechanisms, *Fusarium* infection, Plant growth-promoting rhizobacteria, Sustainable agriculture, Water deficit, *Zea mays* L.

## Abstract

**Main conclusion:**

The review highlights PGPR (e.g., *Pseudomonas* spp.) as sustainable, low-cost solution to mitigate drought and *Fusarium* stress in maize, enhancing yield and resilience.

**Abstract:**

Maize (*Zea mays* L.) is a vital staple crop worldwide, yet its productivity is under growing pressure from the combined effects of drought and *Fusarium verticillioides* infection. These stresses often occur together, compounding the damage. Drought limits water availability, disrupts nutrient uptake, and slows photosynthesis, while also making plants more vulnerable to disease. In turn, *F. verticillioides* harms plant tissues, contaminates grain with *fumonisins*, and can further intensify water stress. Conventional approaches such as irrigation, fungicides, and resistant cultivars often fall short when both stresses occur simultaneously. In recent years, plant growth-promoting rhizobacteria (PGPR), particularly *Pseudomonas* spp., have gained attention as eco-friendly partners in managing these challenges. These beneficial bacteria support maize growth by improving nutrient availability, regulating plant hormones, enhancing osmoprotectants’ production, activating antioxidant defenses, and suppressing pathogens through antifungal compounds, competitive root colonization, and induced systemic resistance. Findings from single-stress experiments show that *Pseudomonas* endophytes can boost drought tolerance by maintaining osmotic balance and antioxidant activity, while also limiting *F. verticillioides* infection and toxin production. However, studies examining their effectiveness under the combined pressures of drought and fungal attack remain limited. This review brings together current knowledge on the mechanisms, case studies, and practical constraints of *Pseudomonas*-mediated stress relief in maize, highlighting research gaps and setting priorities for strain selection, microbial consortia design, and large-scale field testing. Harnessing these bacteria could be a key step toward building climate-resilient maize production systems that protect both yields and grain safety in an era of environmental uncertainty.

**Graphical abstract:**

Exogenous application of *Pseudomonas* spp. modulate drought and *Fusarium verticillioides*.
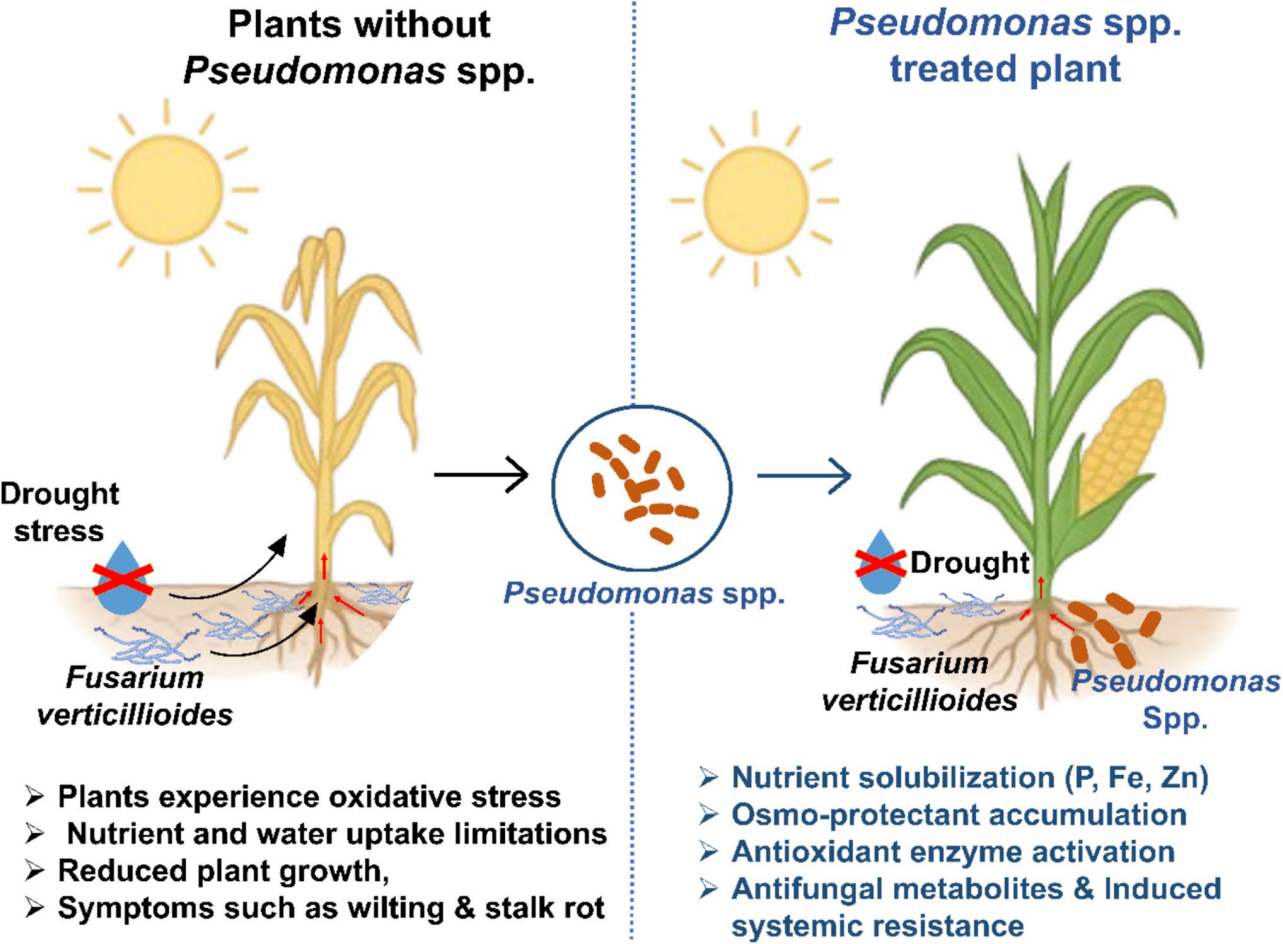

## Introduction

Maize (*Zea mays* L.) is one of the most important cereal crops worldwide, serving as a staple food, livestock feed, and a raw material for numerous industrial applications (Murdia et al. 2016; Amanjyoti et al. 2024; Chukwudi et al. 2025; Deribe 2025). Its high productivity potential and adaptability have made it central to food security in many regions, particularly in sub-Saharan Africa, Latin America, and parts of Asia (Erenstein et al. 2022; Siddique et al. 2022). However, maize production is increasingly threatened by the combined pressures of abiotic and biotic stress factors, with drought (Rahman et al. 2025) and *Fusarium verticillioides* (Omotayo and Babalola 2023) infection emerging as two of the most severe constraints (Ahmadi et al. 2022; Khawula et al. 2023; Deribe 2025).

Drought is a recurrent challenge in maize-growing regions, especially under the influence of climate change, which has intensified the frequency, severity, and unpredictability of water scarcity events (Alattas et al. 2024; Chukwudi et al. 2025). Water scarcity events result in drought stress which is known to impair photosynthetic efficiency, disrupt nutrient uptake, and alter hormonal signaling, resulting in stunted growth and substantial yield losses (Rehman et al. 2023; Rai et al. 2024). More critically, drought stress not only affects plant physiology directly but also predisposes maize to pathogen invasion by weakening host defense mechanisms (Chávez-Arias et al. 2019, 2021; Mirsam et al. 2022; Barzegari and Taghian 2025). Among fungal pathogens, *Fusarium verticillioides* is the causal agent of *Fusarium* ear rot and stalk rot that pose a dual threat to maize production (Deepa et al. 2021; Omotayo and Babalola 2023). Apart from causing physical damage to plant tissues, this pathogen contaminates grains with *fumonisins*, a group of mycotoxins that compromise grain quality and pose significant health risks to humans and livestock (Badiwe 2022; Baard et al. 2023).

In many cases, drought and *F. verticillioides* act synergistically: drought-induced physiological stress creates favorable conditions for fungal colonization, while pathogen infection intensifies plant water stress, leading to accelerated crop decline (Gaige et al. 2020; Mirsam et al. 2022; Tiru et al. 2022). This intersection of abiotic and biotic stresses represents a major hurdle for sustainable maize production (Tiru et al. 2022). Conventional management strategies such as chemical fungicides, irrigation, and resistant cultivars have had limited success under these combined stresses (Pérez-Méndez et al. 2021; Bittencourt et al. 2023; Chauhan et al. 2024). Chemical control raises environmental and health concerns, while irrigation is often economically or logistically unfeasible in drought-prone areas (Baard et al. 2023; Alattas et al. 2024). Moreover, breeding for combined resistance strategies to both drought and fungal pathogens remains a lengthy and complex process (Baard et al. 2023; Kapoor et al. 2024). These limitations highlight the need for innovative, eco-friendly solutions that can enhance plant resilience under multiple stress conditions.

Plant growth-promoting rhizobacteria (PGPR), particularly *Pseudomonas* spp., are a promising biological agent for improving crop productivity and stress tolerance (Mehmood et al. 2023; Sahgal et al. 2024). *Pseudomonas* spp. play a significant role in promoting plant growth and inducing systemic resistance (ISR) against pathogens and other competitive soil microbes. They achieve this through both direct and indirect mechanisms, including the synthesis of phytohormones, and the production of antifungal metabolites and antibacterial proteins, which not only inhibit competing microbes but also facilitate plant–microbe and microbe–plant signaling communication (El-Saadony et al. 2022; Burlakoti et al. 2024).

Importantly, certain strains of *Pseudomonas* have demonstrated the ability to alleviate drought- and biotic stress-induced damage by enhancing root architecture and rapidly colonizing the rhizosphere. This competitive advantage allows them to secure a greater supply of nutrients, thereby depriving other microbes of essential resources, a mechanism that Ali and colleagues linked to suppressing *Fusarium verticillioides* infection (Ali et al. 2022). Despite the growing body of research on PGPR in agriculture, studies explicitly exploring the dual role of *Pseudomonas* spp. in mitigating both abiotic and biotic stress in maize remain limited. This review aims to synthesize current knowledge on the mechanisms through which *Pseudomonas* spp. contribute to growth promotion and stress alleviation in maize, with a particular focus on scenarios involving drought and *F. verticillioides* co-occurrence. It further identifies existing research gaps and outlines potential future directions for integrating *Pseudomonas*-based strategies into climate-resilient maize production systems.

## Drought and *Fusarium verticillioides* stress in maize

### Drought stress in maize plants

Drought is one of the most significant abiotic stresses limiting maize productivity worldwide, particularly in semi-arid and sub-humid regions where rainfall patterns are unpredictable (Deribe 2025; Khurshid et al. 2025). The severity of drought effects depends on the timing, duration, and intensity of the water deficit relative to the crop’s growth stage (Ullah et al. 2024; Yasin et al. 2024). Physiologically, drought reduces plant water potential, disrupts cell turgor, and limits the availability of essential nutrients due to reduced mass flow and diffusion in the soil–root interface (Yasin et al. 2024; Deribe 2025). These changes initiate a cascade of stress responses, including stomatal closure to minimize water loss, reduced leaf expansion, and early senescence (Talukder et al. 2021).

One of the earliest impacts of drought is the decline in photosynthetic activity (Queiroz et al. 2019). Stomatal closure restricts CO_2_ entry into the mesophyll, while water scarcity impairs chloroplast function, leading to reductions in chlorophyll content and photochemical efficiency (Talbi et al. 2020; Zhang et al. 2022). Furthermore, drought induces oxidative stress through the overproduction of reactive oxygen species (ROS) such as hydrogen peroxide (H_2_O_2_), superoxide anions (O_2_^−^), and hydroxyl radicals (OH·), which can damage lipids, proteins, and nucleic acids if not efficiently scavenged by antioxidant systems (Hasanuzzaman et al. 2020; Yasin et al. 2024).

Yield reduction under drought conditions results from a combination of morphological, physiological, and reproductive disruptions. In maize, water scarcity during vegetative stages limits biomass accumulation, while stress during reproductive stages delays silk emergence, reduces pollen viability, and increases the anthesis–silking interval (ASI) (Modhej et al. 2017; Mi et al. 2018). These reproductive impairments often result in poor kernel set, reduced grain filling, and ultimately lower yields (Modhej et al. 2017; Mi et al. 2018). Even mild drought episodes during sensitive growth phases can cause disproportionate yield losses, highlighting the vulnerability of maize to water-limiting conditions (Li et al. 2015).

### *Fusarium verticillioides* mode of infection in maize plants

**Fig. 1:**
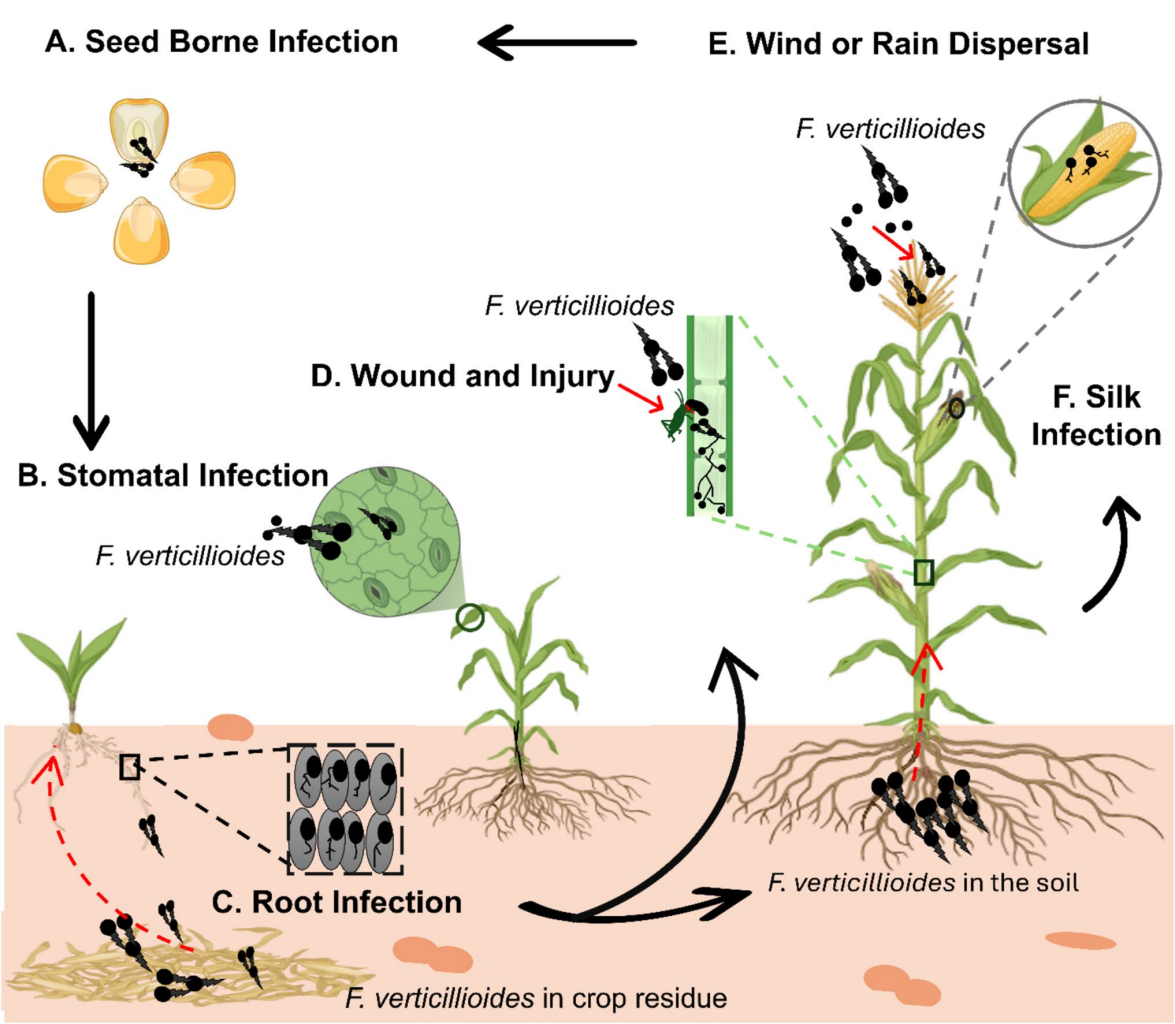
The multiple infection routes of *Fusarium verticillioides* in maize, including seed-borne, stomatal, root, wound-related, silk, and airborne (wind or rain) pathways. These diverse entry points highlight the pathogen’s adaptability and its significant threat to maize growth and yield. The figure was adapted from Xu et al. (2023)

*Fusarium verticillioides* (syn. *Gibberella moniliformis*) is a soil- and seed-borne pathogen that infects maize at multiple stages of development, causing stalk rot and ear rot (Capo et al. 2020). This fungus can survive in crop residues, soil, and infected seeds, allowing it to persist across seasons. *F. verticillioides* employs both biotrophic and necrotrophic strategies to infect its hosts, meaning it can feed off living tissue and later kill it to further its invasion (Gaige et al. 2020). Infection commonly begins through airborne spores that land on the plant, particularly during the flowering (anthesis) stage, when silks and developing panicles are exposed (Fig. 1) (Omotayo and Babalola 2023). Alternatively, soil and seed transmission also serve as major entry points, allowing the pathogen to infiltrate plants during early development (Shaikh 2021; Omotayo and Babalola 2023). Entry is facilitated through natural plant openings such as cracks in the pericarp, pedicel wounds, or mechanical damage (de Sousa et al. 2022). Once inside, the fungus colonizes key plant tissues, such as seeds, roots, stalks, and ears, leading to a variety of diseases (Fig. 1) (Xiong et al. 2024).

Pathogen establishment is often asymptomatic during early infection stages, particularly in root and stalk tissues, but progresses to visible symptoms such as rotting, discoloration, and necrosis (Gai et al. 2018). Ear infection typically occurs through the silks during pollination, especially under humid conditions, and leads to ear rot characterized by white to pink fungal mycelia on kernels (Gai et al. 2018).

### Synergistic effects of drought and *Fusarium verticillioides*

Drought and *Fusarium verticillioides* infection frequently co-occur in maize-growing regions, and it seems as if their interaction often causes more severe damage than either stress alone. Although only a limited number of studies have assessed their combined impact, existing evidence consistently indicates a direct and compounding relationship between the two stresses (Vaughan et al. 2016; Gaige et al. 2020). For instance, Gaige et al. (2020) revealed that drought stress not only facilitated fungal colonization but also intensified its negative impact on seedling growth and biomass accumulation. Vaughan et al. (2016) investigated the interactive effects of elevated CO_2_ and drought stress on maize defense responses against *Fusarium verticillioides*. Their findings showed that elevated CO_2_ alone increased maize’s susceptibility to fungal proliferation, though fumonisin levels per unit of fungal biomass did not rise. However, when elevated CO_2_ was combined with drought stress, the effect was amplified, and maize supported even greater fungal growth and exhibited higher fumonisin contamination due to increased pathogen biomass, although the mycotoxin level relative to fungal mass remained unchanged. These results suggest a synergistic weakening of maize defense systems under simultaneous abiotic stressors, raising significant concerns for food safety in future climate scenarios.

Parsons (2008) examined the biotic and abiotic factors associated with *Fusarium* ear rot of maize and identified insect damage particularly from thrips and corn earworms and drought or inadequate irrigation as strong contributors to increased disease incidence. The study highlighted that the fungus typically infects maize kernels or silks through wounds, often caused by insects, and proliferates most readily during physiological maturity under warm (26–28 °C) or drought stress conditions. These combined environmental and biotic factors facilitate fungal entry, colonization, and subsequent fumonisin accumulation, resulting in yield losses and grain contamination. Together, these studies offer complementary insights into *F. verticillioides*’ epidemiology and impact. Parsons (2008) provides a field-level perspective, mapping out real-world ecological triggers of *Fusarium* ear rot, while Vaughan et al. (2016) deliver mechanistic evidence of how future climatic conditions may exacerbate the problem by altering plant–pathogen interactions.

**Fig. 2:**
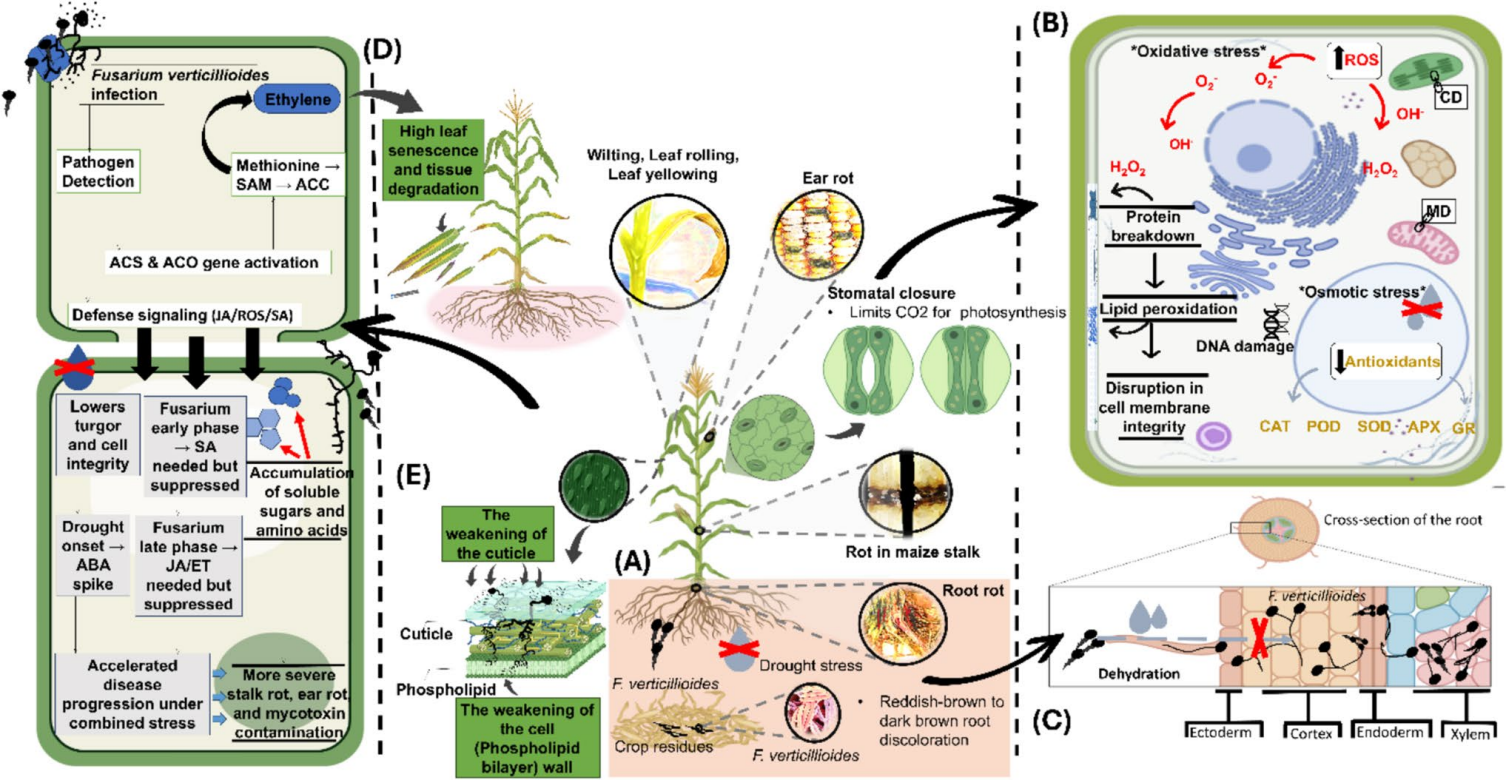
The combined effect of drought stress and *Fusarium verticillioides* infection on maize plants, highlighting both physiological (**A**) and cellular responses (**C**). On the whole-plant level, drought and fungal infection lead to visible symptoms such as wilting, leaf rolling, yellowing, ear rot, stalk rot, and root rot characterized by reddish to dark brown root discoloration. Stomatal closure occurs under drought stress, limiting CO_2_ uptake and thus reducing photosynthesis efficiency. **B** Highlights how at the cellular level, the plant experiences oxidative and osmotic stress, resulting in reactive oxygen species (ROS) accumulation, membrane damage, chloroplast disruption (CD), mitochondrial disruption (MD) and DNA disruption. Due to the combined effect of both stresses, antioxidant enzymes such as CAT (catalase), POD (peroxidase), SOD (superoxide dismutase), APX (ascorbate peroxidase), and GR (glutathione reductase) and their activity is reduced, while **C** shows how the pathogen, often originating from infected crop residues, invades through the roots, particularly under drought-compromised conditions, spreads into the vascular system, disrupting water uptake and causing dehydration and **D** and **E** show ethylene accumulation in plants and defense signaling (JA/SA), respectively. Overall, Fig. 2 highlights how drought and fungal infection together intensify stress and reduce maize productivity

Although current research shows that drought and *Fusarium verticillioides* infection in maize often occur simultaneously, there is still limited understanding of how their interaction influences overall growth and physiological health (Hussain et al. 2019). Drawing from what is already known about the individual effects of these stresses, their combination is likely to be highly damaging. Drought predisposes maize to fungal infection in several ways. First, water deficit weakens structural barriers such as cuticle and cell walls, facilitating pathogen penetration (Zhang et al. 2018; Yasin et al. 2024). Second, drought-induced stomatal closure, while conserving water, can limit the plant’s ability to cool itself, increasing tissue susceptibility to colonization (Devi et al. 2022, 2024; Ghadirnezhad Shiade et al. 2023). Third, drought stress alters hormonal balances such as elevated abscisic acid (ABA) and reduced salicylic acid (SA) that can compromise pathogen defense signaling pathways (Fig. 2) (Agunbiade and Babalola 2023).

From the pathogen’s perspective, drought-related host stress can create favorable conditions for colonization. For example, reduced turgor and cell integrity might allow easier spread within host tissues, while accumulation of soluble sugars and amino acids under drought can serve as nutrient sources for fungal growth (Dastogeer et al. 2019). Moreover, drought-induced reductions in maize’s antifungal metabolites and pathogenesis-related (PR) proteins can lower resistance thresholds (Agunbiade and Babalola 2023).

At the signaling level, the interaction between drought and fungal stress involves complex interplay among ROS, phytohormones, and defense pathways. ROS play dual roles: they serve as signaling molecules that activate defense responses but, when overproduced under drought, can cause oxidative damage that weakens plant tissues (Yang et al. 2019; Yasin et al. 2024). Hormonal interconnection is equally critical. ABA, a key drought-response hormone, can antagonize jasmonic acid (JA) and salicylic acid (SA) pathways that are central to defense against necrotrophic and hemibiotrophic pathogens, including *F. verticillioides*. Conversely, fungal infection can trigger ethylene production, which under drought conditions may increase senescence and tissue degradation (Fig. 2) (Wang 2019; Hassan and Hadi 2022; Sunarti et al. 2022).

### Plant response mechanisms to drought and *Fusarium verticillioides*

Maize plants exhibit a wide array of adaptive mechanisms to withstand drought and *Fusarium verticillioides* infection, involving physiological, biochemical, and molecular responses. While several of these defense strategies overlap between abiotic and biotic stress scenarios, others are uniquely featured to the nature of the stress encountered by maize plants. The efficiency of these responses depends on factors such as the intensity and timing of stress, cultivar genetics, and environmental conditions, with dual-stress situations often imposing competing demands on resource allocation.

### Responses to drought stress

Drought triggers a series of physiological modifications in maize aimed at conserving water and maintaining cellular function. Stomatal closure, regulated primarily by abscisic acid (ABA), reduces transpirational water loss but simultaneously restricts CO_2_ entry into the mesophyll, limiting photosynthetic rates (Yasin et al. 2024). Morphological adjustments, such as leaf rolling, reduced leaf area, and enhanced cuticular wax deposition, further contribute to water conservation (Queiroz et al. 2019; Devi et al. 2022). Root architectural plasticity, characterized by deeper rooting systems and increased lateral root proliferation, enhances access to residual soil moisture, an important trait in drought-resilient genotypes.

Biochemically, drought induces the accumulation of compatible solutes, including proline, glycine betaine, and soluble sugars, which act as osmoprotectants by stabilizing proteins, membranes, and macromolecular structures under dehydration (Hasanuzzaman et al. 2020; Zandi and Schnug 2022). Concurrently, drought stress elevates reactive oxygen species (ROS) such as hydrogen peroxide (H_2_O_2_), superoxide anions (O_2_^−^), and hydroxyl radicals (OH^−^). To counteract potential oxidative damage, maize activates enzymatic antioxidants superoxide dismutase (SOD), catalase (CAT), ascorbate peroxidase (APX) as well as non-enzymatic antioxidants such as ascorbic acid and glutathione (Chrpová et al. 2021; Zandi and Schnug 2022; Kumar 2024; Yasin et al. 2024). At the molecular level, transcription factors such as DREB (dehydration-responsive element binding), NAC (no apical meristem), and bZIP (basic leucine zipper) orchestrate the expression of drought-inducible genes, many of which encode protective proteins (e.g., late embryogenesis abundant proteins) and enzymes involved in osmolyte biosynthesis and ROS detoxification. These responses collectively enhance drought resilience but require significant metabolic investment (Chrpová et al. 2021; Zandi and Schnug 2022; Kumar 2024; Yasin et al. 2024).

### Responses to *Fusarium verticillioides* infection

Defense against *F. verticillioides* begins with preformed barriers, including lignin-rich cell walls, cuticular wax layers, and antimicrobial secondary metabolites that inhibit fungal entry (Omotayo and Babalola 2023). Upon pathogen recognition via pattern recognition receptors (PRRs), maize initiates induced defenses involving both local and systemic responses. Early defense events include the oxidative burst, producing ROS that serve simultaneous roles as antimicrobial agents and signaling molecules to activate downstream defense genes (Naz et al. 2021). This is followed by the synthesis of pathogenesis-related (PR) proteins such as β-1,3-glucanases and chitinases that degrade fungal cell walls, and the production of phenolic compounds and phytoalexins with antifungal activity. Hormonal signaling is central to these defenses (Xu et al. 2022). Jasmonic acid (JA) and ethylene pathways are typically associated with resistance to necrotrophic pathogens, including *F. verticillioides*, whereas salicylic acid (SA) signaling plays a role in early-stage pathogen detection and containment (Quiroz-Figueroa et al. 2023). In addition, induced systemic resistance (ISR) and systemic acquired resistance (SAR) prime uninfected tissues for enhanced defense readiness, reducing the likelihood of secondary infections (Naz et al. 2021).

### Overlapping and unique responses under dual stress

When drought and *F. verticillioides* infection occur concurrently, maize must coordinate responses to both stresses, often under conditions of physiological compromise. Several mechanisms are shared between drought and pathogen defense. For instance, ROS regulation is a unifying theme, as both stresses generate ROS that must be tightly modulated to prevent cellular damage while sustaining their signaling roles (Ramegowda and Senthil-Kumar 2015). Strengthening of cell walls through lignification and callose deposition benefits both drought tolerance and pathogen resistance, while osmolyte accumulation aids in maintaining turgor under drought and may create unfavorable osmotic conditions for fungal proliferation, especially in resistant maize genotypes (Quiroz-Figueroa et al. 2023).

Under combined drought and *Fusarium verticillioides* stress, several interconnected physiological and biochemical processes make maize particularly vulnerable. Drought triggers a rapid increase in abscisic acid (ABA), a key hormone in water stress adaptation, which promotes stomatal closure, osmotic adjustment, and activation of drought-responsive genes. However, this comes at a cost, as elevated ABA can antagonize the jasmonic acid (JA) and salicylic acid (SA) pathways that are central to activating plant defenses against pathogens, thereby reducing resistance to *F. verticillioides* (Ollas and Dodd 2016; Ali et al. 2020; Muhammad Aslam et al. 2022; Mishra 2023). In parallel, the plant reallocates significant metabolic resources such as carbohydrates, ATP, and reducing power toward drought survival strategies, including osmotic regulation and cell wall reinforcement, leaving fewer resources for the synthesis of pathogenesis-related (PR) proteins and antifungal secondary metabolites (Ollas and Dodd 2016; Rafique 2020; Chávez-Arias et al. 2021). Physiologically, drought also accelerates leaf senescence and tissue desiccation, weakening structural barriers and releasing nutrients that favor necrotrophic pathogens like *F. verticillioides* (Rafique 2020). These senescing tissues provide both entry points and a conducive microenvironment for fungal proliferation (Nephali et al. 2021; Agunbiade and Babalola 2023). Collectively, hormonal antagonism, resource allocation trade-offs, and structural weakening under drought create conditions that not only facilitate *F. verticillioides* colonization but also heighten the risk of *fumonisin* contamination, posing a dual threat to maize’s yield and food safety (Chávez-Arias et al. 2019).

While these mechanisms provide strong evidence of how drought may enhance *F. verticillioides* infection and *fumonisin* contamination, the precise extent to which these stresses interact, affect overall maize growth, yield, and quality under field conditions remains poorly understood. Experimental studies that integrate physiological, biochemical, and agronomic measurements under combined stress scenarios are still limited, leaving uncertainty in predicting real-world outcomes, especially under variable climate conditions. In addition, the complex interplay between abiotic and biotic stress responses highlights the difficulty in breeding or engineering maize varieties with dual resistance. This complexity also highlights the potential value of biological interventions such as *Pseudomonas* spp. capable of modulating multiple stress-response pathways simultaneously, thereby providing integrated protection against drought and fungal pathogens (Bittencourt et al. 2023; Alattas et al. 2024).

## *Pseudomonas* spp. as plant growth-promoting rhizobacteria (PGPR)

### *Pseudomonas* spp. diversity and taxonomy

The genus *Pseudomonas* comprises a diverse group of Gram-negative, rod-shaped, motile bacteria belonging to the family *Pseudomonadaceae*. Members of this genus are ubiquitously distributed in soil, water, and plant-associated environments, with several species well documented for their plant growth-promoting traits and antagonistic activity against phytopathogens (Rajkumar et al. 2024). While certain species, such as *Pseudomonas aeruginosa*, are recognized as opportunistic human pathogens, non-pathogenic strains of *P. aeruginosa* and other species including *Pseudomonas fluorescens*, *Pseudomonas putida*, *Pseudomonas chlororaphis*, and *Pseudomonas protegens* are widely studied for agricultural applications (Sandhya et al. 2017; Omotayo and Babalola 2023; Ameen et al. 2024).

### General PGPR traits

*Pseudomonas* spp., as plant growth-promoting rhizobacteria (PGPR), exhibit multiple functional traits that contribute to enhanced crop growth, yield, and resilience against biotic and abiotic stresses (Alattas et al. 2024; Sahgal et al. 2024). A key mechanism is nutrient solubilization, whereby these bacteria convert insoluble forms of essential nutrients such as phosphorus (P), iron (Fe), and zinc (Zn) into plant-available forms (Hyder et al. 2023; Kapoor et al. 2024). Phosphate-solubilizing *Pseudomonas* strains release organic acids and phosphatases that mobilize phosphorus, while siderophore production facilitates iron acquisition by chelating Fe^3^⁺ and making it accessible to plants under iron-limiting conditions. Similarly, zinc-solubilizing activity improves micronutrient uptake, thereby supporting enzyme activity and metabolic processes (Alattas et al. 2024). In addition to nutrient mobilization, *Pseudomonas* spp. modulates phytohormones, producing indole-3-acetic acid (IAA) to stimulate root elongation, cytokinins to promote cell division and shoot growth, and enzymes such as ACC deaminase to regulate ethylene levels, thus mitigating stress-induced growth inhibition (Voronina et al. 2023; Kapoor 2024). Collectively, these PGPR traits contribute to improved plant establishment, nutrient acquisition efficiency, and tolerance to environmental stressors, underscoring their potential as sustainable bioinoculants in modern crop production systems (Kálmán et al. 2024; Kapoor et al. 2024; Khurshid et al. 2025).

### Adaptability and rhizosphere competence

The ecological success of *Pseudomonas* spp. as PGPR is largely attributed to their ability to rapidly colonize root surfaces and persist in the rhizosphere even under adverse conditions. Being known as prominent and efficient colonizers, they play a role in early protection and promoting enhanced growth. Although these bacterial endophytes constitute phenomenal benefits, a study by Barea et al. (2005) and Kumar et al. (2011) reported that the colonization strength of *Pseudomonas* spp. strains deteriorates quickly than those from *Bacillus* and *Trichoderma* species. Their inability to produce spores as the Gram-negative rhizobacteria thereby caused it to have a short lifespan (Kumar et al. 2011). However, *Bacillus* sp. are Gram-positive and capable of synthesizing endospores, which ultimately promote a steady and more effective colonization, able to last for years in associated root zones. Similarly, *Trichoderma* is an endophytic fungi that can stay active in associated roots for an entire growing season using the chlamydospores and impressive mycelial growth (Waghunde et al. 2016; Natsiopoulos et al. 2024).

**Fig. 3:**
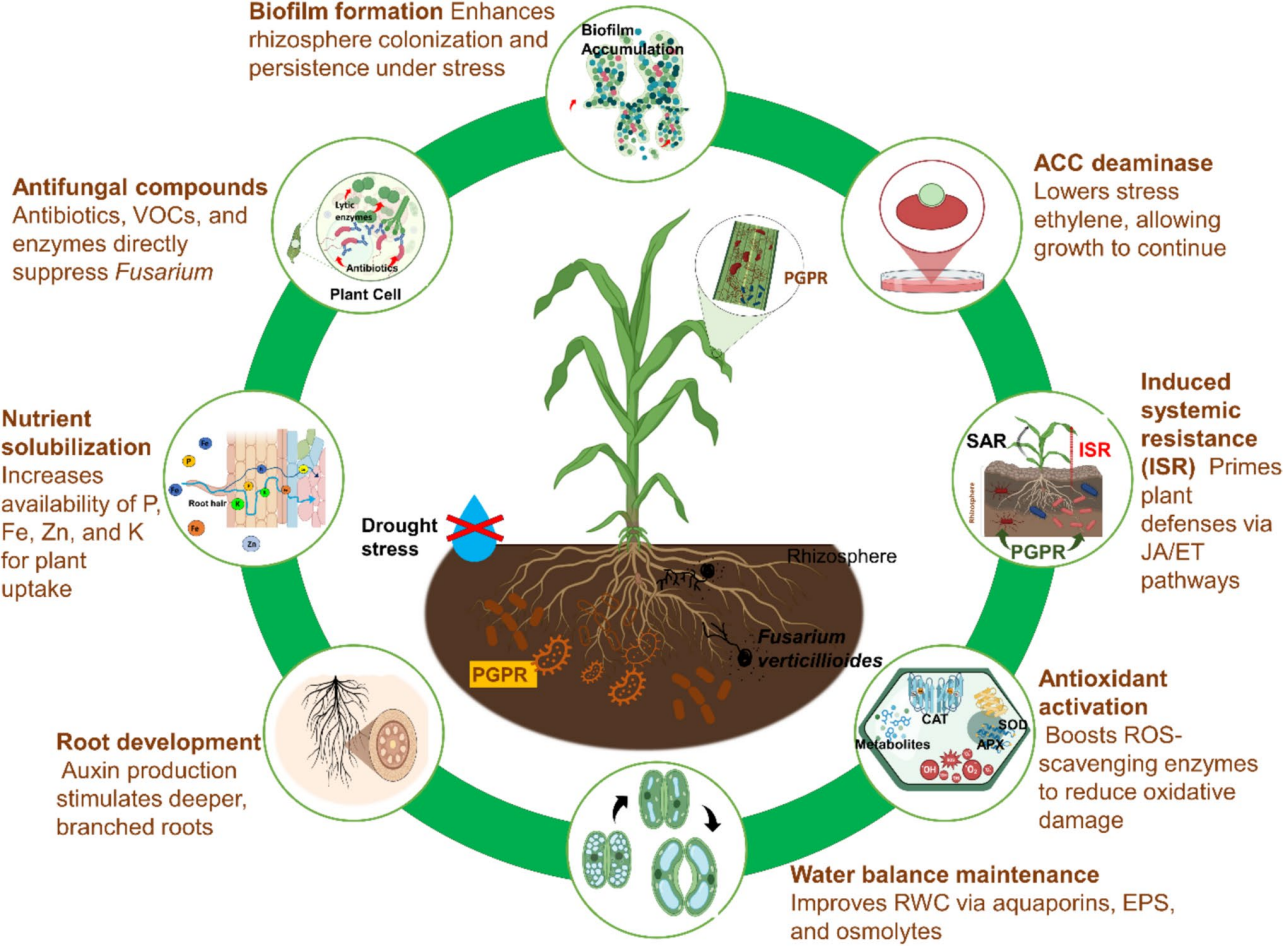
Integrated mechanisms by which *Pseudomonas* spp. may enhance maize tolerance to combined drought and *Fusarium verticillioides* stress. The schematic illustrates eight complementary modes of action: ACC deaminase reduces stress-induced ethylene, induction of systemic resistance (ISR), antioxidant activation such as catalase (CAT), superoxide dismutase (SOD), ascorbate peroxidases (APX), water balance maintenance, root development, nutrient solubilization, antifungal compounds including antibiotics, volatile organic compounds (VOCs), and lytic enzymes, may directly suppress *F. verticillioides*, and biofilm formation

Despite this, *Pseudomonas* sp. are still the better choice for annual crops like maize, due to their ability to provide an immediate colonization, attributing to an early primed plant defense in developing root tips (Alattas et al. 2024). Many strains from *Pseudomonas* genera produce biofilms, which not only facilitate strong root attachment but also provide protection against desiccation and environmental fluctuations (Zboralski and Filion 2020). Quorum sensing (QS) systems regulate the production of secondary metabolites and biofilm formation, enabling these bacteria to modulate their behavior in response to plant and microbial signals (Fig. 3) (Santoyo et al. 2021). Genomic and transcriptomic analyses have revealed that *Pseudomonas* spp. possess large genomes enriched with genes for secondary-metabolite biosynthesis, efflux pumps, and stress tolerance, suggesting a broad capacity to interact with and adapt to diverse plant hosts and stress conditions (Dorjey et al. 2017; Çiftçi et al. 2023; Mehmood et al. 2023).

## Case studies and experimental evidence

### Case study 1: endophytic *Pseudomonas* enhancing drought tolerance in maize

A study by Sandhya et al. (2010), in a greenhouse experiment, drought-tolerant *Pseudomonas* strains were inoculated onto maize seedlings under controlled water-deficit conditions. Treated plants exhibited improved growth parameters, higher leaf relative water content, increased accumulation of osmolytes such as proline and soluble sugars, and elevated activities of antioxidant enzymes (e.g., superoxide dismutase and catalase). These physiological changes collectively enhanced plant adaptation to drought stress (Fig. 3).

### Case study 2: *Pseudomonas* spp. suppressing *Fusarium* spp. in maize

A field and in vitro study evaluated the antagonistic potential of *Pseudomonas fulva* PS9.1 against *F. verticillioides*. The strain significantly inhibited fungal growth and reduced disease severity in maize, likely through production of antifungal metabolites and competition for rhizosphere colonization sites. This demonstrated the potential of *Pseudomonas* as part of an integrated *Fusarium* management program (Adeniji and Babalola 2022).

### Case study 3: microbial consortia with *Pseudomonas* providing root protection against *F*. *verticillioides*

In a rhizosphere microbiome manipulation study, synthetic microbial communities enriched with *Pseudomonas* spp. were shown to provide superior protection to maize roots against *F. verticillioides* compared to single-strain inoculations. The consortia enhanced root colonization, suppressed fungal proliferation, and improved overall plant vigor, highlighting the potential benefits of cooperative microbial interactions (Niu et al. 2017).

### Case study 4: field-based evidence linking beneficial microbes to drought and disease tolerance in maize

A review by Backer et al. (2018) of multiple field trials found that rhizosphere- and endophyte-associated *Pseudomonas* strains improved maize performance under both drought-prone and disease-conducive environments, primarily by modulating phytohormones, improving nutrient uptake, and activating induced systemic resistance (ISR). Though they provide protection for an entire plant growth life cycle, the allocation of resources and defense-related mechanisms is prioritized according to the plant’s developmental stage (Kálmán et al. 2024). Notably, at the seedling phase, PGPR contributes to early crop establishment and reduces crop failure by depending on plant’s ability to biosynthesize phytohormones, which support vegetative and reproductive phases, and also improve its adaptation response (Ansary et al. 2012), whereas the matured plants rely more on rhizospheric competence and survival ability, together with their metabolic activity, to strengthen weak immunity and enhance resistance against late-season root and stalk rot (Lastochkina et al. 2020).

Although nothing has been documented on maize under these stresses, other studies, such as that of Lastochkina et al. (2020), support this notion, where water-stressed wheat plants infected with *Fusarium* root rot (FRR) and inoculated with *Bacillus* sp. exhibited an upregulation in growth parameters and elevated resistance from both stress factors. While not always tested, and with lack of evidence on maize crops under combined stress, the few studies that evaluated these effects collectively confirmed broad-spectrum resilience benefits.

Individually, studies relating to these stresses demonstrate that *Pseudomonas* endophytes and rhizobacteria can provide multiple, well-documented benefits to maize under stress conditions. For instance, certain strains enhance drought tolerance by promoting osmotic adjustment, strengthening antioxidative defense systems, and maintaining favorable plant water status, as shown in case study 1. Other strains are effective in suppressing *Fusarium verticillioides* through the production of antifungal metabolites, competitive exclusion in the rhizosphere, and improvements in root health, as highlighted in case studies 2 and 3. Beyond targeting specific stresses, *Pseudomonas* spp. can also promote broad-spectrum plant resilience by activating induced systemic resistance (ISR) and improving nutrient acquisition, as outlined in case study 4. Collectively, these mechanisms suggest that *Pseudomonas* endophytes have the potential to support maize’s performance across diverse environmental challenges (Fig. 3). Taken together, these findings provide strong mechanistic and experimental evidence that *Pseudomonas* endophytes can help maize tolerate each stress independently.

Although *Pseudomonas* demonstrates efficacy in promoting plant growth when applied as a single strain, the use of PGPR consortia has yielded better results across multiple crop species (Saleem et al. 2021). Numerous consortia of *Pseudomonas* sp. in their individual complements or in collaboration with other PGPR strains such as *Bacillus* and *Trichoderma* sp. showed successful facilitation of enhanced resilience against drought and *Fusarium* diseases even under field level (Akhtar et al. 2018; Chukwudi et al. 2025). For example, Saleem et al. (2021), who investigated this under drought conditions, reported that a PGPR consortium (*B. pumilus* and *P. putida*) significantly uplifted maize yield (6.278 t/ha). Therefore, this forwards a strong suggestion that synthetic consortia of *Pseudomonas* sp. have the potential to perform well under dual-stress conditions, considering the strains’ complementary function. Logically, the drought-primed physiological improvements (e.g., water retention and ROS scavenging) could create a stronger defense environment against *Fusarium* infection, while the pathogen-suppressive traits could reduce biotic damage when plants are already water-stressed. However, direct studies that impose both drought and *Fusarium* stresses on maize while testing endophytic *Pseudomonas* as a treatment are currently rare or absent in the literature. This gap highlights the need for future field trials explicitly designed for multi-stress scenarios, which could validate the promising dual benefits suggested by these single-stress case studies.

## Mechanistic pathways of *Pseudomonas* spp.-mediated stress mitigation in maize

The ability of *Pseudomonas* spp. to alleviate stress in maize is rooted in their multifaceted physiological, biochemical, and molecular interactions with the plant. These mechanisms can be classified according to their primary target stress drought, *Fusarium verticillioides* infection, or their combined occurrence, though significant overlap exists due to the complexity of plant–microbe–environment interactions (Zboralski and Filion 2023). The next section of this review will explore in detail the mechanisms of *Pseudomonas*-mediated stress alleviation, focusing on how these bacteria modulate plant physiology, enhance tolerance to drought, and suppress *F. verticillioides* under individual stresses, predicting how these responses might be impacted under combined stress conditions (Fig. 3).

### Drought-specific mechanisms

Under water-deficit conditions, *Pseudomonas* spp. primarily mitigate stress by modulating plant water relations and enhancing osmotic adjustment. Many strains synthesize and stimulate plant production of osmoprotectants such as proline, glycine betaine, and soluble sugars, which maintain cell turgor during dehydration (Aslanpour et al. 2016; Lucas et al. 2023). The production of exopolysaccharides (EPS) by rhizobacteria improves soil aggregation and water retention in the rhizosphere, thereby extending moisture availability to roots (Hyder et al. 2023; Kapoor et al. 2024). In addition, the secretion of 1-aminocyclopropane-1-carboxylate (ACC) deaminase reduces plant ethylene levels, preventing premature senescence and root growth inhibition. Induction of antioxidant enzymes, including superoxide dismutase (SOD), catalase (CAT), and peroxidases (POD), counteracts drought-induced reactive oxygen species (ROS) accumulation, protecting cellular structures from oxidative damage (Kálmán et al. 2024; Chukwudi et al. 2025).

### *Fusarium verticillioides*-specific mechanisms

The antagonistic activity of *Pseudomonas* spp. against *F. verticillioides* involves direct pathogen suppression and the fortification of host defenses. Antibiosis is achieved through the secretion of secondary metabolites such as phenazines, pyoluteorin, and hydrogen cyanide (HCN), which inhibit fungal growth and sporulation (Ayala-Torres et al. 2023). Competitive exclusion occurs when *Pseudomonas* colonizes root surfaces and niches, limiting the pathogen’s access to infection sites (Stanojković-Sebić et al. 2017). Siderophore production deprives the fungus of iron, an essential micronutrient for its metabolism (Stanojković-Sebić et al. 2017; Lv et al. 2023). On the host side, *Pseudomonas*-induced systemic resistance (ISR) primes maize defense pathways, leading to elevated expression of pathogenesis-related (PR) proteins, strengthening of cell walls through lignin deposition, and accumulation of phytoalexins, thereby reducing fungal penetration and colonization (Lv et al. 2023). Studies evaluating this phenomenon under in vitro experiment observed that *Pseudomonas* isolates can significantly inhibit the growth of *Fusarium* spp., including *F. verticillioides*, through the production of antifungal compounds and competitive exclusion (Eraky and Gomaa 2012; Stanojković-Sebić et al. 2017).

In the case of contaminated maize cultivars, some endophytic bacterial strains have demonstrated strong inhibitory effects against *Fusarium* spp., resulting in reduced infection severity and lower fumonisin accumulation. Mishra et al. (2022) reported that *Pseudomonas fluorescens* strain JM-1, which produces 2,4-diacetylphloroglucinol (DAPG), exhibited pronounced antagonism toward *Fusarium moniliforme*. This activity was attributed to the production of antifungal metabolites that suppressed ear rot development and fumonisin biosynthesis in maize kernels, thereby enhancing grain quality and yield. Beyond direct inhibition of fungal growth, certain PGPR strains can also modulate the expression of *Fusarium* genes responsible for toxin synthesis. Pereira et al. (2011) observed that maize treated with antagonistic bacterial strains exhibited reduced expression of the *FUM1* gene and lower fumonisin B1 (FB1) levels (Bacon et al. 2001), even though fungal mycelial growth was not significantly affected. However, these beneficial effects are often influenced by maize genotype, kernel developmental stage, and prevailing environmental conditions. Such variability may explain why the same *Pseudomonas* strain performs effectively in one setting but less so in another. Consequently, when selecting PGPR strains for use as biofertilizers or bioinoculants, it is crucial to assess their consistency, stability, and biocontrol efficacy under environmental conditions similar to those of the intended application site.

### Dual-stress mitigation mechanisms

Since studies which directly examine the role of *Pseudomonas* spp. in maize under simultaneous drought and *Fusarium verticillioides* infection remain limited, it is likely that these bacteria could induce an integrated protective response discussed above to address both these abiotic and biotic stresses. Previous research has shown that *Pseudomonas* strains enhance drought tolerance by producing ACC deaminase and osmoprotectants, which help maintain water balance and reduce stress-induced ethylene production (Danish et al. 2020; Chandwani and Amaresan 2024). Separately, *Pseudomonas fluorescens* and related species have demonstrated the ability to suppress fungal pathogens, including *Fusarium* spp., by inducing systemic resistance and producing antifungal metabolites (Fig. 3) (Chandra Nayaka et al. 2009; Noumavo et al. 2015; Amein and Fatah 2022; Mishra et al. 2022).

Endophytic bacteria from the *Pseudomonas* genus possess the capacity to serve as crucial biological modulators of phytohormonal interaction between abscisic acid (ABA), central to drought adaptation, and jasmonic acid/ethylene (JA/ET), vital in pathogen defense. Such modulation might mitigate the antagonistic signaling often observed between these pathways, thereby promoting coordinated activation and improved tolerance under combined abiotic and biotic stresses. Under combined stresses, plants inoculated with *Pseudomonas* sp. are highly likely to shift their defense responses toward an induced systematic resistance (ISR). A JA/ET-dependent pathway enables protection against pathogens related diseases, while also combating the negative effects of drought stress. Cho et al. (2013) conducted a transcriptome analysis on *Arabidopsis thaliana* plants, inoculated with *P. chlororaphis* O6 strain, grown under drought-induced conditions.

Their observation revealed that non-stressed plants showed increased levels of the genes associated with Jasmonic (VSP1 and pdf-1.2) and salicylic (PR-1) acid responsive pathway (JA/SA), while transcription factors related to ethylene (ET) and abscisic acid (ABA) signaling pathway decreased in non-colonized *thaliana plants*. However, the opposite case was noted in water-stressed plants inoculated with O6 strain, and results showed an upregulation of genes (HEL) responsible for boosting disease-resistance genes, indicated in clusters; however, less of HEL was found in the control. Although direct investigations under combined drought and *Fusarium* infection specifically focusing on maize plants are lacking, these findings provide a credible basis to hypothesize that *Pseudomonas* spp. may coordinate dual tolerance mechanisms, mitigating oxidative damage, maintaining physiological balance, and limiting fungal colonization. To confirm this, targeted studies exploring *Pseudomonas*-mediated responses under concurrent drought and *Fusarium verticillioides* stress in maize are urgently needed.

## Limitations/challenges

While these insights highlight the promising potential of *Pseudomonas* spp. in mitigating both drought and *Fusarium verticillioides* stresses, their application in real-world agricultural systems is not without challenges. Variability in strain performance across environments, inconsistencies in field efficacy compared to controlled conditions, and gaps in our understanding of their behavior under combined stresses outline the need for a critical appraisal of their limitations. Reviewing these constraints is essential, not only to set realistic expectations for their deployment but also to guide targeted research and development toward strains and application strategies that can deliver consistent, durable benefits in the field (Table 1).

**Table 1:** Challenges and limitations of *Pseudomonas* spp. in maize stress mitigation

Category	Challenge/limitation	Reference
Drought stress	Inconsistent efficacy across different maize genotypes and environmental conditions	A study found that the effectiveness of *Pseudomonas* spp. in enhancing drought tolerance varied among different maize genotypes and environmental conditions, indicating the need for genotype-specific and environment-specific applications (Notununu et al. 2024). Similarly with *Pseudomonas* biofilms, they can be relatively stable under transient soil moisture fluctuations because the total exopolysaccharide (EPS) (e.g., alginate and Psl/Pel) buffers cells against desiccation and osmotic stress, and root-associated microhabitats further mitigate water loss. However, their stability is strain-dependent and conditioned by EPS genetics, the frequency and severity of drying/rewetting cycles, soil texture, and host root exudation; repeated or extreme moisture fluctuations may promote biofilm turnover or shifts in community composition (Chang et al. 2007; Marshall et al. 2019; Craig et al. 2021; Bhattacharyya et al. 2023; Bolin et al. 2023)
	Potential negative interactions with native soil microbiota	Research highlights that the introduction of *Pseudomonas* spp. into the soil could disrupt existing microbial communities, potentially leading to unintended ecological consequences (Glandorf et al. 2001; Orlewska et al. 2018; Birnbaum et al. 2023)
*Fusarium verticillioides*	Might have limited persistence and colonization in the rhizosphere	*Pseudomonas* spp. face competition from other rhizosphere microorganisms, such as Bacillus and Burkholderia, which also exhibit antagonistic properties against *F. verticillioides*. This competition can limit their ability to establish and persist in the rhizosphere (Sameer et al. 2018; Gaige et al. 2020; Omotayo and Babalola 2023). The effectiveness of *Pseudomonas* spp. is influenced by environmental factors such as soil type, moisture, and nutrient availability, which can affect their growth and colonization ability (Gaige et al. 2020)
	Variability in antifungal activity among different *Pseudomonas* strains	Research demonstrates that not all *Pseudomonas* strains exhibit the same level of antifungal activity against *Fusarium verticillioides*, suggesting that strain selection is crucial for effective biocontrol (Stanojković-Sebić et al. 2017). For instance, antifungal secondary-metabolite production is highly strain-dependent and also responsive to environmental context; phenazines, pyoluteorin, and hydrogen cyanide (HCN) are common in fluorescent pseudomonads but their expression and quantities vary widely between strains and with growth environment. Culture and rhizosphere surveys and functional studies report both presence/absence of biosynthetic genes and quantitative variation in metabolite levels; several field and rhizosphere studies document phenazine-producing strains in cereal rhizospheres but emphasize spatial, genotypic and edaphic variability. Direct quantitative in-planta measurements remain fewer than in vitro screens, so careful strain selection and in-field metabolite profiling are recommended (Woeng et al. 2003; Gilchrist et al. 2011; Yuan et al. 2020; Shahid et al. 2021)
Dual-stress conditions	Lack of comprehensive studies combining drought and *Fusarium* stress	There is scarcity of studies investigating the combined effects of drought and *Fusarium verticillioides* on maize, highlighting a significant research gap in understanding the interaction between these stress factors. However, in other cereal crops like rice, the application of *P. fluorescens* SP007s demonstrated enhance resistance against common soil pathogen diseases and water stress via an activation of defense-related enzymes such as POX, PAL, GPX, and SOD when drought was induced for 14 days (after 90-day of growth) (Prathuangwong et al. 2012)
General limitations	Regulatory and commercial challenges in adopting microbial inoculants	There is a lack of standardized protocols for the production and quality control of microbial inoculants, which affects their reliability and efficacy in the field including *Pseudomonas* spp. The regulatory environment for microbial inoculants is often underdeveloped, leading to inconsistencies in product approval and market entry across different regions (de Sousa et al. 2022; Fadiji et al. 2024; Díaz-Rodríguez et al. 2025)

## Research gaps and future perspectives

Identifying research gaps is essential for guiding future studies toward areas where current knowledge remains limited or inconsistent. In the context of *Pseudomonas*-mediated stress mitigation, this PGPR shows strong potential for enhancing tolerance to combined drought and *Fusarium* stresses. However, addressing these gaps is critical to unlocking its full potential and paving a clear path forward.

## Limited understanding of dual-stress interactions

Although *Pseudomonas* spp. have been extensively studied for mitigating either drought or *Fusarium verticillioides* stress individually, there remains a significant lack of research exploring their efficacy under simultaneous dual-stress conditions. Maize plants in the field frequently face combined abiotic and biotic stresses, which can interact in complex and sometimes unpredictable ways (Parsons 2008; Leitão et al. 2020). These combined stresses can modulate plant physiological and molecular responses differently than single-stress factors, necessitating comprehensive investigations into how *Pseudomonas* spp. influence plant resilience in such multifaceted environments (Leitão et al. 2020; Chávez-Arias et al. 2021). Future research should develop integrated experimental models that simulate concurrent drought and pathogen stresses to better replicate field conditions and uncover synergistic or antagonistic effects on maize growth and defense response.

## Variability in microbial performance and host specificity

The inconsistent colonization, persistence, and functional efficacy of *Pseudomonas* strains in different soil types, maize genotypes, and environmental conditions remain major limitations (Mosimann et al. 2017; Chukwudi et al. 2025). Not all strains exhibit robust antifungal activity or drought mitigation capabilities, which constrains the development of universally effective bioinoculants (Ðalovic et al. 2013). Therefore, there is a pressing need for systematic screening and selection of *Pseudomonas* strains tailored to specific agroecological contexts and maize varieties. For instance, Saleem et al. (2021) investigated the effects of several PGPR strains, including *Pseudomonas fluorescens* 1, on two maize cultivars differing in drought tolerance: NK-6654 (drought-tolerant) and SD-626 (drought-sensitive). The same *Pseudomonas* strain elicited contrasting responses between the two genotypes. Under water-stressed conditions, inoculated NK-6654 plants showed significant improvements in water uptake and photosynthetic efficiency, whereas SD-626 plants exhibited a marked decline in these parameters. These findings highlight that the growth-promoting efficiency of *Pseudomonas* spp. is not always consistent across genotypes and can be strongly influenced by the host’s genetic adaptability and the prevailing environmental conditions.

## Insufficient mechanistic insights into stress interactions

Previous studies have demonstrated that abscisic acid (ABA) functions antagonistically with the jasmonic acid (JA) and salicylic acid (SA) signaling networks during combined abiotic and biotic stress responses (Gupta et al. 2017; Long et al. 2019). However, there remains a huge gap regarding how *Pseudomonas* spp. influences plant hormonal signaling, particularly pathways mediated by abscisic acid (ABA) and jasmonic acid/ethylene (JA/ET), especially in maize plants. It would be interesting for future research to investigate well-known *Pseudomonas* spp., such as *Pseudomonas putida* KT2440, and *Pseudomonas fluorescens* WCS374, which have been shown to upregulate ABA-related genes in roots while inducing certain JA-pathway responses (Neal and Ton 2013; Planchamp et al. 2015; Wang et al. 2020) suggesting a capacity to impact both abiotic and biotic stress signaling, however, with differences in tissue specificity and timing, and isolates like *Pseudomonas argentinensis* SA190 capable of inducing systemic resistance (ISR) through JA/ET pathways (Cordero et al. 2012; Lafi et al. 2016; Chu et al. 2018; Holečková et al. 2018). However, direct evidence of *Pseudomonas* simultaneously enhancing both ABA- and JA/ET-dependent responses under combined stresses in maize is limited, with most existing work focusing on one pathway or stress type. This gap highlights a promising avenue for further investigation into how these species influence such mechanisms and whether specific *Pseudomonas* strains can overcome the antagonistic hormonal interplay that often limits plant performance under simultaneous abiotic and biotic challenges.

To address this gap, a targeted experimental framework could be designed. This would involve selecting a set of well-characterized strains, including a known ISR inducer such as *P. fluorescens* WCS374, a metabolically versatile rhizosphere colonizer such as *P. putida* KT2440, and a drought-adapted isolate such as *P. argentinensis* SA190 (Cordero et al. 2012; Neal and Ton 2013; Lafi et al. 2016; Chu et al. 2018; Holečková et al. 2018). Plants could be exposed to individual stresses (drought or *Fusarium verticillioides* infection) as well as a combined stress regime (drought plus *Fusarium verticillioides*). Hormonal profiling quantifying ABA, JA, and ET concentrations along with expression analysis of key marker genes in both roots and shoots should be conducted at multiple time points. Physiological assessments such as stomatal conductance, wilting scores, and survival rates, alongside disease severity indices, would provide integrated insights into stress tolerance. This way, the aim would be to identify strains that either (a) simultaneously enhance ABA and JA/ET signaling, (b) induce JA/ET without altering ABA, or (c) elevate ABA while repressing JA/ET. Such comparative outcomes would directly test whether certain *Pseudomonas* strains can harmonize hormonal interaction to improve plant resilience under combined abiotic and biotic stresses.

## Overlooked importance of the seedling stage under dual stresses

A notable and often underappreciated research gap lies in the insufficient focus on the seedling stage of maize when investigating combined drought and *Fusarium verticillioides* stresses. Most existing studies concentrate on mature plants or individual stress factors, leaving the early developmental phase relatively understudied in dual-stress contexts. This is a critical oversight because the seedling stage is arguably the most vulnerable period in the crop lifecycle, where initial root establishment, water uptake, and pathogen defense are crucial for subsequent growth and yield potential (Akinwale et al. 2016; Hussain et al. 2019, 2020; González-Hernández et al. 2021).

Seedlings exposed simultaneously to drought and pathogen pressure face compounded challenges; water deficit impairs root development and function, while pathogen infection can cause early mortality or stunted growth, severely limiting the plant’s capacity to withstand later stresses (Akinwale et al. 2016; Hussain et al. 2019; González-Hernández et al. 2021). Moreover, the efficacy of *Pseudomonas* spp. as biocontrol and growth-promoting agents at this stage might have long-lasting impacts on plant health, yet few or no studies have systematically evaluated microbial inoculation during germination and early seedling growth under combined stresses.

Understanding how *Pseudomonas* spp. modulates seed germination, root architecture, early antioxidant responses, and hormone signaling under dual stress is vital for developing targeted seed treatments or soil inoculants. Effective management at the seedling stage can improve crop establishment, reduce seedling mortality, and set the foundation for enhanced tolerance throughout the crop’s life cycle. Addressing this gap will enable the development of more precise, stage-specific microbial interventions, which are essential for integrated stress management in maize cultivation under increasingly unpredictable environmental conditions.

## Integration with other methods

Studies on the integration of *Pseudomonas* spp. with agricultural fungicide with an aim to reduce the overuse of chemical treatments in maize crops remain scarce. This integration might be feasible but context-dependent. Reports show both antagonism and compatibility between (various) microbial inoculants and fungicides; compatibility depends on fungicide mode of action, formulation, timing of application and microbial formulation; some studies report successful co-applications or sequential regimes that preserve inoculant viability and improve control. Combining biologicals with resistant cultivars is conceptually complementary (additive or synergistic disease suppression), and field studies combining *Pseudomonas* strains with host resistance or other biocontrols have shown benefit, but interactions should be tested case by case (Imperiali et al. 2017; Ons et al. 2020; Santos et al. 2021).

In the years ahead, the real strength of *Pseudomonas fluorescens* may lie in how well it works alongside other allies in plant protection. Pairing it with *Trichoderma* spp., for instance, has already proven effective in suppressing *Fusarium oxysporum* by blocking the enzymes the pathogen uses to break down plant cell walls (Nepali et al. 2018). Similarly, building mixed communities where *Pseudomonas* strains are integrated with other beneficial bacteria has been shown to improve their ability to take hold and thrive in a variety of maize genotypes (Pinisetty et al. 2022). Harnessing these partnerships could unlock new, effective disease control strategies that are not only more robust but also better equipped to adapt to the unpredictable challenges of real-world farming.

## Importance of multi-environment testing

One of the key limitations in current *Pseudomonas*-mediated stress mitigation research is the inconsistency of results when applied under different environmental conditions (Mehmood et al. 2023; Zboralski and Filion 2023). While most studies are conducted in controlled greenhouse or laboratory environments, these settings do not fully reflect the diversity of soils, climates, and agricultural practices encountered in real farming systems (Mehmood et al. 2023; Zboralski and Filion 2023). Greenhouse results with *Pseudomonas* biocontrol or growth promotion are often predictive but not fully reproducible across sites because field efficacy is influenced by soil type, native microbiome, climate, crop variety, and agronomic practice. Meta-analyses and direct comparisons show many strains that perform well in controlled conditions lose or show variable efficacy in multi-location field trials; conversely, some strains translate successfully when formulated properly and evaluated across seasons (Imperiali et al. 2017; Tienda et al. 2020; Kashyap et al. 2023). We therefore treat greenhouse data as an essential screening step, but emphasize the need for multi-site, multi-season validation and reporting of soil and climatic metadata.

Therefore, it is essential to evaluate *Pseudomonas* inoculants through coordinated, multi-location trials across varied regions and soil types. Such testing will reveal how environmental factors influence their performance, generate robust data on yield improvement, stress tolerance, and microbial survival, and ensure that recommendations are both reliable and region-specific for farmers.

## Regulatory and commercialization challenges

There are demonstrable ecological considerations when introducing non-native or high-titer microbial inoculants: potential outcomes include transient displacement of native taxa, altered nutrient-cycling functions, or unintended suppression of beneficial organisms. Studies show introduced strains may persist, interact chemically with natives, or be outcompeted, with outcomes varying with strain traits and environment (McSpadden Gardener 2007; Juhanson et al. 2009; Wang et al. 2024; Velte et al. 2025). Regulatory frameworks (varies by country) require risk assessment, environmental fate and non-target impact studies for commercial microbial products. Since quantitative, long-term, multi-site assessments of inoculant impacts on native soil microbiomes are still scarce for many agricultural *Pseudomonas* strains, we highlight this as a research and regulatory priority and suggest pre-release environmental impact studies (persistence, horizontal gene transfer potential, functional assays) as part of responsible deployment.

The regulatory landscape governing the approval and commercialization of microbial bioinoculants remains underdeveloped in many regions, limiting their adoption (Batista and Singh 2021). Clear guidelines, quality standards, and risk assessments are needed to build confidence among stakeholders, including farmers, industry, and policymakers (O’Callaghan et al. 2022). Moreover, economic analyses demonstrate that the cost-effectiveness and sustainability benefits of *Pseudomonas*-based products will support wider market uptake. Collaborative efforts involving researchers, regulatory agencies, and the private sector are therefore essential to streamline the pathway from laboratory research to commercial application (Singh et al. 2021; Mehmood et al. 2023; Thakur et al. 2023).

By addressing these key research gaps through interdisciplinary and collaborative approaches, the scientific community can unlock the full potential of *Pseudomonas* spp. to enhance maize resilience against the increasingly prevalent combined stresses of drought and *Fusarium verticillioides*, contributing significantly to global food security in a changing climate.

## Conclusions and recommendations

This review highlights the promising potential of *Pseudomonas* spp. as versatile plant growth-promoting rhizobacteria capable of enhancing maize resilience against drought and *Fusarium verticillioides* stresses. Through diverse mechanisms such as nutrient solubilization, phytohormone modulation, antifungal metabolite production, and induced systemic resistance, these beneficial bacteria can mitigate both abiotic and biotic challenges that limit maize productivity. However, the effectiveness of *Pseudomonas* spp. is influenced by strain specificity, environmental conditions, and complex interactions within the plant rhizosphere, highlighting the need for targeted research and development. To fully harness the benefits of *Pseudomonas*-based bioinoculants, future research should prioritize integrated studies that reflect real-world dual-stress scenarios, combining drought and pathogen pressures. Advances in molecular biology and omics technologies can clarify the underlying interactions between plant, microbe, and environment, enabling the design of robust microbial consortia tailored to specific agroecological contexts. In addition, extensive multi-location field trials are essential to validate laboratory findings and optimize application strategies for diverse farming systems.

Addressing regulatory and commercialization challenges is equally important to facilitate the adoption of *Pseudomonas* inoculants by farmers. Clear policies, quality control standards, and economic incentives will help translate scientific innovations into sustainable agricultural practices. Collaborations among researchers, industry stakeholders, and policymakers will be critical to overcoming these barriers. In conclusion, *Pseudomonas* spp. represent a sustainable and eco-friendly tool to improve maize tolerance to combined drought and *Fusarium verticillioides* stress, contributing to enhanced crop productivity and food security under climate change. Strategic investments in multidisciplinary research, technology transfer, and stakeholder engagement will be pivotal to realizing this potential on a larger scale.

## Data Availability

This review article does not include new data, software, or code. All information discussed is derived from previously published sources, which are appropriately cited in the manuscript. As such, no datasets or materials are available for sharing.
