# Correction: The potential of *Pseudomonas* spp. as sustainable bioinoculants for enhancing maize growth and integrated management of drought and *Fusarium verticillioides* stress

**DOI:** 10.1007/s00425-026-04943-x

**Published:** 2026-02-21

**Authors:** Khethiwe Ndlazi, Siyabonga Ntshalintshali, Lungelo Buthelezi, Ashwil Klein, Marshall Keyster, Mbukeni Nkomo, Arun Gokul

**Affiliations:** 1 Department of Agriculture, University of Zululand, Main Road, P/Bag X1001, Kwadlangezwa, 3886 South Africa; 2 Plant Biotechnology Laboratory, School of Science and Agriculture, Westville Campus, University of KwaZulu-Natal, University Road, Durban, 4000 South Africa; 3 School of Biology and Environmental Sciences, Faculty of Agriculture and Natural Sciences, University of Mpumalanga, Mbombela, 1200 South Africa; 4 Plant Omics Laboratory, Department of Biotechnology, University of the Western Cape, Robert Sobukwe Road, Bellville, 7530 South Africa; 5 Environmental Biotechnology Laboratory, Department of Biotechnology, University of the Western Cape, Robert Sobukwe Road, Bellville, 7530 South Africa

**Correction: Planta (2025) 263:58** 10.1007/s00425-025-04906-8

In this article, Arun Gokul was incorrectly denoted as the corresponding author but it should have been Mbukeni Nkomo.

In this article, the affiliation details for Arun Gokul were incorrectly given as ‘Department of Plant Sciences, Qwaqwa Campus, University of the Free State, Kestell Road, Phuthaditjhaba 9866, South Africa’ but should have been ‘School of Biology and Environmental Sciences, Faculty of Agriculture and Natural Sciences, University of Mpumalanga, Mbombela, 1200, South Africa’; Arun.Gokul@ump.ac.za.

The original article has been corrected.

